# Role of oregano and *Citrus* species-based essential oil preparation for the control of coccidiosis in broiler chickens

**DOI:** 10.1186/s40104-021-00569-z

**Published:** 2021-04-06

**Authors:** Francisco Xavier Gordillo Jaramillo, Da-Hye Kim, Sang Hyeok Lee, Sun-Kwan Kwon, Rajesh Jha, Kyung-Woo Lee

**Affiliations:** 1 Department of Animal Science and Technology, Konkuk University, 120 Neungdong-ro, Gwangjin-gu, Seoul, 05029 Republic of Korea; 2 Harim Bio Research Center, Jeilfeed Co., LTD, 136 Techno2-ro, Yuseong-gu, Deajeon-si, 34025 Republic of Korea; 3 Department of Human Nutrition, Food and Animal Sciences, College of Tropical Agricultural and Human Resources, University of Hawaii at Manoa, Honolulu, HI 96822 USA

**Keywords:** Broiler chickens, Coccidiosis, Essential oils, Growth performance, Salinomycin

## Abstract

**Background:**

Due to presence of drug-resistant *Eimeria* strains and raised public health safety concerns about drug residues in the meat, there is renewed interest in the search for natural alternatives to the coccidiosis control agents. This study was conducted to test the anticoccidial efficacy of oregano and *Citrus* spp.-based essential oils for broilers.

**Methods:**

A total of 280 7-day-old broiler chicks were fed a control diet or diets with salinomycin or essential oils for up to 35 d of age. On d 14, half of the control groups and the treated groups were orally challenged with a coccidiosis vaccine at 25 times higher than the recommended vaccine dose. Control diet-fed chickens that were gavaged with phosphate-buffered saline were considered non-challenged control group.

**Results:**

*Eimeria* challenge or dietary additives failed to affect growth performance during the 7 to 20 d growth period although essential oil-fed chickens exhibited the lowest body wight gain (*P* = 0.332) and the highest feed conversion ratio (*P* = 0.062). Oocysts in the litter were detected in the challenged control diet group and the challenged/essential oil-fed groups at 21 and 35 d, respectively. Superoxide dismutase activity in the serum was elevated (*P* = 0.059) in the salinomycin-fed chickens compared to the challenged controls. Alpha-1-acid glycoprotein was decreased by 28.7% in the salinomycin-fed chickens but increased by 38.1% in the essential oil group compared with the challenged control group. Challenged control group exhibited a significantly higher cooking loss of the thigh meat, compared to the non-challenged control diet group, which was marginally mitigated by dietary supplementation with essential oils. Chickens fed essential oil-added diet had the highest branched-chain fatty acids contents in the cecum.

**Conclusions:**

In conclusion, this study shows that oregano and *Citrus*-based essential oil preparation did not affect growth performance in broiler chickens challenged with the coccidiosis vaccine nor did *Eimeria*-specific duodenal lesion. However, dietary essential oil preparation lowered oocysts present in litter materials and altered branched-chain fatty acids in cecal digesta. Beneficial findings of the essential oil preparation on volatile fatty acids and oocysts output may warrant further research into assessing its effectiveness and its efficacy in pathogenic field-isolate *Eimeria* spp.-induced coccidiosis disease model.

## Background

Avian coccidiosis is a devastating enteric disease that causes considerable economic loss to the poultry industry worldwide, accounting for US $3 billion annually. This loss is mainly due to prophylactic or therapeutic in-feed medications and compromised health of the afflicted chickens [1]. Avian coccidiosis is caused by several species of *Eimeria*, which are infectious protozoa that penetrate and damage the epithelial cells of the intestinal tissue, resulting in inflammation and hemorrhage [2]. The intestinal damage decreases feed intake, retards growth, and suppresses humoral- and cell-mediated immune responses, all of which adversely affect the poultry industry [3]. Avian coccidiosis was typically controlled by anticoccidial drugs such as synthetic (chemical) and ionophoric feed additives [4]. However, the long-term use of anticoccidial agents has led to drug-resistant *Eimeria* strains and raised public health safety concerns about drug residues in the meat [5]. Thus, there is renewed interest in the search for natural alternatives to the coccidiosis control agents [6, 7] and others like effective vaccines [4, 5]. Essential oils, classified as phytogenics [8], are a mixture of aromatic substances from herbs and spices. They have been used as an alternative to in-feed antibiotics in monogastric animals due to their antimicrobial properties [9]. The inclusion of essential oils in broilers’ diets stimulated growth, modulated host immunity, improved gut health, and increased meat quality [8]. Essential oils from *Origanum vulgare* contain thymol and carvacrol as dominant components and have been widely used in poultry feed due to their strong antimicrobial and anticoccidial activities [10]. Essential oils from *Citrus* species, including orange, lemon, and lime contain limonene, linalool, and r-terpinene as dominant components and have been used as flavoring agents in the food and perfume industries [11]. The *Citrus*-derived essential oils have also been used as food supplements to inhibit growth of food-poisoning pathogens [12] and as a feed additive for chickens [9]. *Citrus*-based essential oils also exhibit anthelminthic [13] and antiparasitic activities [14]. Essential oils from *O. vulgare* and *Citrus* species have anticoccidial activities, so the synergistic effects of their combined preparations may provide a natural solution to control avian coccidiosis [15]. Indeed, a blend of essential oils from *O. vulgare* and *Citrus* spp. exhibited anticoccidial activities by decreasing coccidia invasion and stimulating growth in lambs [16]. Thus, it was hypothesized that a blend of *O. vulgare* and *Citrus* spp.-based essential oils may be an effective alternative to control avian coccidiosis. To test the hypothesis, we reproduced experimental avian coccidiosis with high doses of the coccidiosis vaccine (as demonstrated by [17, 18]). As far as we know, this study was the first to test the anticoccidial activities of a blend of *O. vulgare* and *Citrus* spp.-based essential oils in coccidiosis chicken disease model.

## Materials and methods

### Experimental design, animals, and diets

A total of 280 1-day-old male broiler chicks (Ross 308) were obtained from a local hatchery. Upon arrival, the chicks were individually weighed and randomly placed into 28 floor pens with fresh rice hulls as a bedding material (10 chicks/pen, 5 chicks/m^2^) and provided a commercial pre-starter diet. The corn and soybean meal-based pre-starter diet was in a crumbled form and fed from 1 to 7 d of age. The pre-starter diet had 3025 kcal metabolizable energy per kg of diet and 22% crude protein. Seventy additional chicks were housed separately in the floor pens and used to replace dead or culled chicks during the first week.

**Fig. 1:**
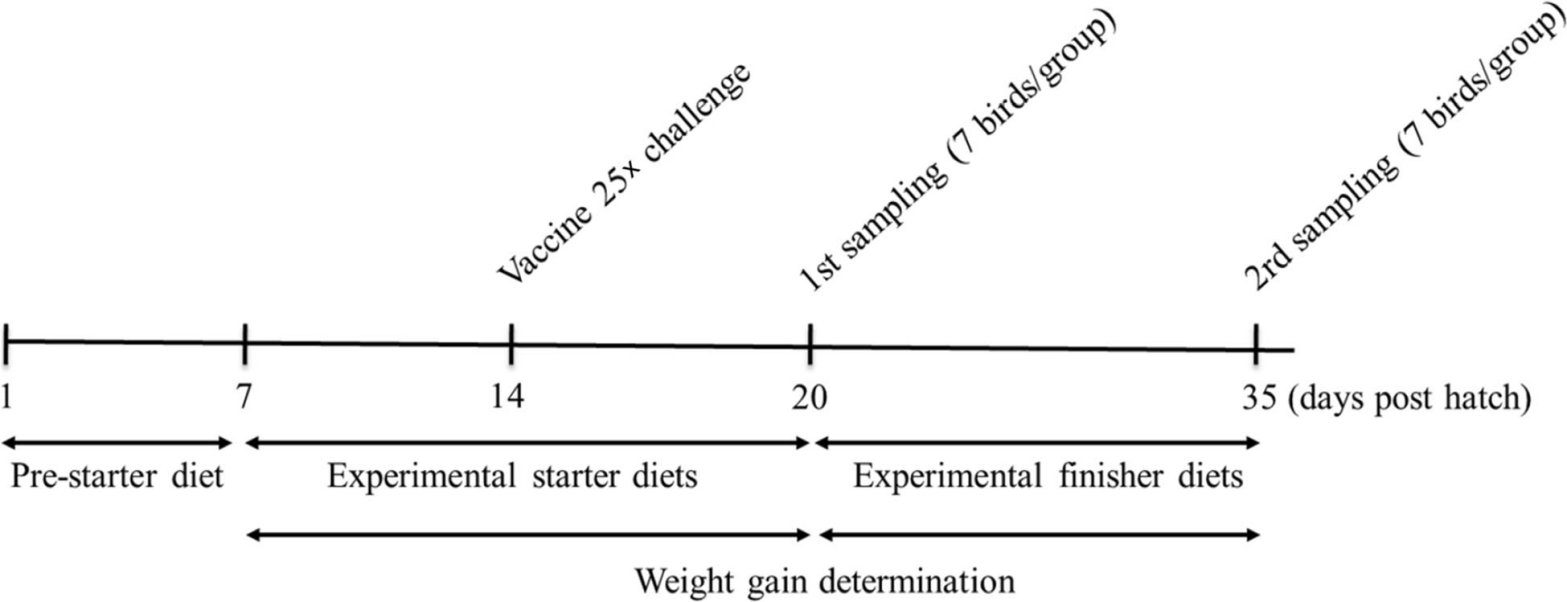
Schematic outline of the experimental schedule. The experimental diets were provided from 7 d post-hatch until the end of the experiment. Chickens were orally challenged with 25× the recommended dose of the coccidiosis vaccine at d 14. Blood, intestine, and meat samples were obtained on d 20 and 35, and body weights were assessed at 7, 20, and 35 d

In a completely randomized design, the chicks (on d 8) weighing (on average) 191 ± 0.4 g/bird per pen were randomly assigned to one of the following diets: a non-supplemented control diet or diets supplemented with salinomycin (SAL; 60 mg/kg of diet; Decoxan®, Korea THUMB VET, Jeonbuk, South Korea) or an essential oil (EO) preparation (500 mg/kg of diet). Additives were thoroughly mixed in the basal feeds before use. The commercially available EO preparation has been marketed as the natural alternative to coccidiostats and is a blend of essential oils extracted from oregano (*Origanum vulgare*) and *Citrus* spp., as described earlier [16]. Dietary EO was added at the concentration of 500 mg/kg of diet, of which inclusion level has been known to alleviate avian coccidiosis [10]. At d 14, half of the control group (challenged control; INFECT) and all of the treated groups were challenged with 25× the recommended dose (5 × 10^4^ oocysts per bird) of the live coccidiosis vaccine (Coccivac®-D, Intervet/Merck Animal Health, Omaha, NE, USA). We selected the 25× manufacturer’s recommended dose of the live coccidiosis vaccine in an attempt to induce mild to moderate coccidiosis without mortality [18]. In addition, the authors chose the challenge time point at d 14 posthatch so that the anticoccidial effect of the tested additives could be determined on d 20 onward by measuring various parameters such as *Eimeria*-specific gut lesions, oocyst output, and growth performance. Per the manufacturer’s specification, the vaccine contains live oocysts of *E. acervuline, E. mivati, E. maxima, E. tenella, E necatrix, E. brunetti, E. hagani, and E. praecox*. The remaining control group chicks were gavaged with phosphate-buffered saline (CON; i.e., the non-challenged control). Each treatment had seven replicates of 10 chicks each (*n* = 70 chicks/treatment). The experimental schedule is depicted in Fig. 1.

The chicken facility was initially set at 32 °C, but the temperature was gradually decreased to 25 °C at week 3 and maintained at this temperature thereafter. The light cycle was one-hour of darkness per day. Corn and soybean meal-based starter and finisher diets (Table 1) were formulated. The starter diet was provided from d 8 to 20, after which the finisher diet was provided until the end of the experiment (35 d). The basal diets did not contain antibiotics or anticoccidials but included a non-starch polysaccharide degrading enzyme (Hemicell®, Elanco, Greenfield, IN, USA), phytase enzyme (Quantum® Blue, ABVista, Wiltshire, UK), and alternative to antibiotics (Monolaurin, Silo S.P.A., Firenze, Italy).

**Table 1:** Ingredients and nutrients composition of the starter and finisher diets

Ingredients, g/100 g	Starter (8–20 d)	Finisher (21–35 d)
Corn	42.96	42.32
Wheat	23.00	25.00
Soybean meal	27.41	25.46
Corn gluten meal	1.00	1.00
Beef tallow	2.50	2.50
Lysine HCl	0.11	0.72
MHA^a^ (liquid methionine)	0.44	0.45
*L*-Threonine	0.09	0.08
Salt	0.24	0.25
Limestone	1.21	1.18
DCP^a^	0.63	0.63
Vitamin/mineral premix^b^	0.25	0.25
Choline chloride	0.05	0.05
NSPase^a^ enzyme	0.05	0.05
Phytase	0.01	0.01
AGP^a^ alternative	0.05	0.05
Total	100	100
Chemical composition		
Metabolic energy^c^, kcal/kg	3150	3200
Dry matter^d^, %	87.8	87.9
Crude protein^c^, %	20.0	19.0
Crude fat^d^, %	5.34	4.85
Crude ash^d^, %	5.14	4.79
Calcium^c^, %	0.80	0.78
Total phosphorus^c^, %	0.54	0.49
Available phosphorus^c^, %	0.35	0.35
Lysine^c^, %	1.33	1.22
Methionine^c^, %	0.70	0.70

^a^*MHA* Methionine hydroxy analogue, *DCP* Dicalcium phosphate, *NSP* Non-starch polysaccharide, *AGP* Antibiotic growth promoter

^b^Vitamin and mineral premix provided the following nutrients per kg of diet: vitamin A, 19,000 IU; vitamin D_3_, 5000 IU; vitamin E, 50 mg; vitamin K_3_, 5.25 mg; thiamine, 3.50 mg; riboflavin, 14 mg; pyridoxine, 7 mg; cobalamin, 0.027 mg; niacin, 146.0 mg; biotin, 0.21 mg; folic acid, 1.75 mg; pantothenic acid, 22.85 mg, Fe,72 mg; Mn, 90 mg; Zn, 74 mg; I, 1.8 mg; Se, 0.36 mg; Cu, 4.8 mg

^c^Calculated values

^d^Analzyed values

### Sampling

Feed intake and body weight per pen were measured at 8, 20, and 35 d post-hatch and used to calculate the body weight gain (BWG), feed intake and feed conversion ratio (FCR). Mortality was recorded daily and used to adjust the growth performance data. On d 20, one bird per pen was randomly selected and euthanized by carbon dioxide (CO_2_) asphyxiation. Immediately after euthanasia, blood was collected in vacutainer tubes by heart puncture, serum was obtained by gentle centrifugation (at 200×*g* for 15 min), and the samples were stored at − 20 °C until further use. Immediately after blood sampling, the small intestine and a pair of ceca were excised. A segment of the duodenum was processed for measurements of the *Eimeria*-specific lesion and secretory immunoglobulin A (sIgA). The ceca were kept on ice until preparation for measurements of the volatile fatty acids (VFA) (described below). On d 35, one bird per pen was randomly selected and euthanized as described above. Immediately after euthanasia, the breast and thigh meats were sampled for meat quality. Finally, to monitor oocyst counts in the litter, five 500 g subsamples from 5 different spots (3 subsamples around the nipples and 2 subsamples around the feeder) in each pen were sampled on d 21 and 35.

### Gut lesion scores and oocyst counts in the litter

On 6 d post coccidiosis vaccine challenge (d 20 posthatch), the lesion score was measured on the duodenum of the small intestine [18]. However, it should be noted that coccidiosis vaccine used in this study contained multiple *Eimeria* species that can affect various segments of intestine ranging from duodenum to ceca. Duodenum was selectively examined for *Emeria*-induced lesions to evaluate the anticoccidial effects of the tested additives. The severity of the lesion was scored on a scale of 0 to 4 by three observers blind to the experimental treatments. Total oocysts present in the litter samples were isolated as described earlier [19] and oocysts isolated were counted using McMaster chamber. Oocysts were expressed as log oocyst counts per g of litter.

### Secretory immunoglobulin A (sIgA) contents in the gut mucosa

On d 20, the duodenal segments were opened and washed to remove the digesta. The mucosa was collected by gently scraping the gut surface, homogenized with ice-cold sterilized saline, and centrifuged at 4000 r/min for 10 min. The supernatant was stored at − 20 °C until further use. The secretory immunoglobulin A (sIgA) contents were measured via commercially available quantitative enzyme-linked immunosorbent assay (ELISA) kits (Bethyl Laboratories, Montgomery, TX, USA) according to the manufacturer’s recommendations. The sIgA contents are expressed per gram of protein. The protein content of the supernatant was measured by a commercial BCA Assay kit (Merck, Darmstadt, Germany).

### Volatile fatty acids

Approximately 1 g of cecal digesta was added to 9 mL of cold distilled water and homogenized on an Ultra-Turrax (Digital Ultra-Turrax T25, IKA, Staufen, Germany). The mixture was combined with 0.05% of saturated mercuric chloride (HgCl_2_), 1 mL of 25% phosphoric acid (H_3_PO_4_), and 0.2 mL of 2% pivalic acid (as an internal standard) and centrifuged at 1000 × *g* at 4 °C for 20 min. The supernatant (1.5 mL) was collected and stored at − 20 °C until analysis. The concentrations of VFA were measured by gas chromatography (6890 Series GC System, HP, Palo Alto, CA, USA) as described by Kim et al. [20].

### Serum parameters

The serum samples were analyzed for nitric oxide by using the Griess reagent (Sigma, St. Louis, MO, USA), glutathione by using the QuantiChorm antioxidant Assay Kit (BioAssay Systems, Hayward, CA, USA), superoxide dismutase (SOD) by using the SOD determination kit (Sigma, St. Louis, MO, USA), and alpha-1-acid glycoprotein by using the chicken alpha-1-acid Glycoprotein Assay Kit (Life Diagnostics Inc., West Chester, PA, USA). All assays were conducted per the manufacturer’s guidelines.

### Meat quality

The breast and thigh muscles were evaluated for meat quality parameters (cooking loss, pH, and color 24 h postmortem). To measure the cooking loss, fresh meats were placed in individual vacuum-sealed plastic bags, immersed in a water bath at 80 °C for 30 min, and cooled in running tap water (on ice) for 20 min. The residual moisture from each sample was absorbed with tissue/filter paper. The cooking loss was calculated as the difference between the uncooked and cooked weights. The pH values were measured at three locations using a portable pH meter (Testo 205, AG Germany). The color of the fresh meats, including the lightness (L*), redness (a*), and yellowness (b*) values, was measured using a reflectance colorimeter (CM-2600d/2500d, Minolta, Japan). Color was measured in triplicate on the bone-side surface of each sample, and the colorimeter was calibrated with a standard white ceramic tile.

### Statistical analysis

All of the data were analyzed by one-way analysis of variance using the general linear model procedure in SAS V9.3 (SAS Institute Inc., Cary, NC, USA). If the F-tests for treatment effects were significant, the differences between treatment means were tested by Duncan’s multiple range test. The pen was considered an experimental unit. The significance level was pre-set at *P* < 0.05, and tendency was declared at *P* < 0.10.

## Results

There were no differences (*P* > 0.05) in BWG between the CONT and INFECT groups at any age, though BWG in the former was 4.80% higher, on average, during d 7 to 20 (Table 2). The addition of SAL to the broilers’ starter diet (7 to 20 d) tended to increase BWG by 7.0% compared to the INFECT group. On the other hand, dietary EO did not affect BWG. None of treatments affected feed intakes. The FCR was highest (*P* = 0.062) in the EO group, followed by the INFECT group, and was lowest in the CON and SAL groups. Both BWG and FCR during the finisher (21 to 35 d) and whole grow-out (7 to 35 d) periods were similar to the trends observed for 7 to 20 d. In short, chickens in the CON and SAL groups grew faster compared to the EO-added diet-fed chickens although there were no statistical differences between groups. Similarly, the FCR was higher in the INFECT and EO groups, but lower in the CON and SAL groups during the finisher phase (*P* = 0.054) and whole grow-out periods (*P* = 0.057).

**Table 2:** Effects of the dietary essential oil preparation on the growth performance in coccidiosis vaccine-challenged broiler chickens^a^

Item	Treatments^b^				SEM	*P*-value
	CON	INFECT	SAL	EO		
7 to 20 d						
BWG, g/bird	678.7	646.1	677.8	633.9	19.916	0.332
Feed intake, g/bird	1073	1024	1065	1064	22.350	0.629
Feed to gain, g/g	1.590	1.609	1.576	1.705	0.032	0.062
21 to 35 d						
BWG, g/bird	1493	1463	1490	1441	40.452	0.793
Feed intake, g/bird	2462	2480	2325	2418	67.659	0.396
Feed to gain, g/g	1.641	1.698	1.566	1.701	0.037	0.054
7 to 35 d						
BWG, g/bird	2147	2100	2167	2088	50.764	0.678
Feed intake, g/bird	3486	3471	3388	3479	74.620	0.797
Feed to gain, g/g	1.625	1.671	1.568	1.688	0.031	0.057

^a^Values represent the mean of 7 replicates per treatment (*n* = 7/treatment)

^b^*CON* Non-challenged naïve control, *INFECT* Challenged control, *SAL* Challenged/salinomycin-added diet, *EO* Challenged/essential oil-added diet, *BWG* Body weight gain, *SEM* Standard error of the means

*Eimeria*-specific lesions on the duodenal segments were not observed in the CON group but appeared in all chickens of the challenged groups (i.e., INFECT, SAL, and EO groups) at 6 d post-challenge. There were no differences in the gut lesions between the INFECT and challenged/treated groups (i.e., SAL and EO; Table 3). The INFECT control had the highest oocyst counts per g of litter (log_10_ 2.97), followed by the EO group (log_10_ 2.87) on d 21 (Table 3). However, no oocysts were detected in the SAL-fed group. On d 35, oocysts were detected in all of the challenged groups, but there were significantly more oocysts (*P* < 0.05) in the INFECT group than in the EO-treated group. No oocysts were present in the litter samples of the CON group (at any age).

**Table 3:** Effects of the dietary essential oil preparation on the duodenal lesion scores and oocyst counts per gram of litter^1^

Item	Treatment^2^				SEM	*P*-value
	CON	INFECT	SAL	EO		
d 20						
Lesion score	0^b^	1.86^a^	1.71^a^	1.86^a^	0.233	< 0.001
Oocysts, log_10_ oocysts/g litter						
d 21	0^b^	2.97^a^	0^b^	2.87^a^	0.183	< 0.001
d 35	0^c^	3.15^a^	2.82^ab^	2.77^b^	0.114	< 0.001

^1^Values represent the mean of 7 replicates per treatment (*n* = 7/treatment)

^2^*CON* Non-challenged naïve control, *INFECT* Challenged control, *SAL* Challenged/salinomycin-added diet, *EO* Challenged/essential oil-added diet, *SEM* Standard error of the means

^a-c^Means with different superscripted letters differ significantly (*P* < 0.05)

*Eimeria* challenge marginally reduced (*P* = 0.059) the SOD activity in the serum samples compared to the CON group (Table 4). However, supplementation of SAL or EO into the broilers’ diets increased the *Eimeria*-mediated decrease in serum SOD activity, being the former vs. the latter more effective. *Eimeria* challenge or dietary interventions did not affect the serum concentrations of glutathione or nitric oxide. The serum alpha-1-acid glycoprotein level at d 20 was 160% higher in the INFECT group than in the CON group. The SAL-fed chickens had lower alpha-1-acid glycoprotein concentrations but the EO-fed chickens had higher concentrations (compared to the INFECT group). The sIgA contents in the duodenal mucosa were 2.4–3.5 ng/g of protein and did not differ (*P* > 0.05) between treatments (data not shown).

**Table 4:** Effects of the dietary essential oil preparation on the serum antioxidant and immunity markers in coccidiosis vaccine-challenged broiler chickens^1^

Item	Treatment^2^				SEM	*P*-value
	CON	INFECT	SAL	EO		
d 20						
SOD activity, %	96.2	94.3	96.4	95.0	0.540	0.059
Glutathione, μmol/L	139.0	136.3	135.3	138.3	1.384	0.241
α1-AGP, μg/mL	211.8^b^	551.1^ab^	392.7^b^	760.8^a^	101.09	0.026
Nitric oxide, μmol/L	19.8	21.1	17.2	19.7	1.245	0.254

^1^Values represent the mean of 7 replicates per treatment (*n* = 7/treatment)

^2^*CON* Non-challenged naïve control, *INFECT* Challenged control, *SAL* Challenged/salinomycin-added diet, *EO* Challenged/essential oil-added diet, *SOD* Superoxide dismutase, *α1-AGP* Alpha-1-acid glycoprotein, *SEM* Standard error of the means

^a-b^Means with different superscripted letters differ significantly (*P* < 0.05)

It was observed that acetate was the dominant VFA in the cecum, followed by butyrate and propionate (Table 5). Branched-chain fatty acid (BCFA) contents (i.e., isobutyrate, isovalerate, and valerate) were kept low in all treated groups and their concentrations were less than 0.8 μmol/g cecal contents. Although non-significant (*P* = 0.164), *Eimeria* challenge lowered the total VFA contents by 19% compared to the CON group. The EO group had highest contents (*P* < 0.05) of isobutyrate and BCFA in the cecum.

**Table 5:** Effects of the dietary essential oil preparation on the concentrations of cecal volatile fatty acids (μmol/g) on d 20 in coccidiosis vaccine-challenged broiler chickens^1^

Item^1^	Treatment^2^				SEM^3^	*P*-value
	CON	INFECT	SAL	EO		
Acetate	44.3	36.4	39.1	42.6	4.190	0.560
Propionate	3.38	2.98	2.24	3.73	0.357	0.106
Isobutyrate	0.16^ab^	0.15^b^	0.09^b^	0.24^a^	0.027	0.021
Butyrate	16.5	12.5	14.8	17.6	2.620	0.590
Isovalerate	0.30	0.28	0.19	0.53	0.079	0.061
Valerate	0.62	0.61	0.62	0.79	0.117	0.693
BCFAs	1.12^b^	1.02^b^	0.72^b^	1.81^a^	0.161	0.003
Total VFAs	65.3	52.9	57.0	65.5	8.784	0.164

^1^Values represent the mean of 7 replicates per treatment (*n* = 7/treatment)

^2^*CON* Non-challenged naïve control, *INFECT* Challenged control, *SAL* Challenged/salinomycin-added diet, *EO* Challenged/essential oil-added diet, *BCFAs* Branched-chain fatty acids (isobutyrate + valerate + isovalerate), *Total VFAs* Total volatile fatty acids (acetate + propionate + butyrate + isobutyrate + valerate + isovalerate), *SEM* Standard error of the means

^a-b^Means with different superscripted letters differ significantly (*P* < 0.05)

*Eimeria* challenge or dietary additives did not affect the meat yields and qualities of broiler chickens sampled at 35 d (Table 6). *Eimeria* challenge increased the cooking loss of the thigh meats compared to the CON group. However, dietary supplementation with SAL or EO did not counteract the challenge-induced increase in cooking loss, although the latter partially decreased it.

**Table 6:** Effects of the dietary essential oil preparation on the yields and quality of breast and thigh meats from coccidiosis vaccine-challenged broiler chickens^1^

Item	Treatment^2^				SEM	*P*-value
	CON	INFECT	SAL	EO		
Breast meat						
Meat, g/100 BW	8.40	8.36	8.06	8.28	0.226	0.735
Cooking loss, %	17.6	17.3	16.0	17.3	0.633	0.385
CIE L* (lightness)	54.2	56.0	56.1	54.7	1.366	0.713
CIE a* (redness)	0.117	−0.344	−0.914	−0.297	0.354	0.258
CIE b* (yellowness)	10.9	10.4	10.2	10.3	0.600	0.836
pH	5.65	5.72	5.63	5.62	0.031	0.198
Leg meat						
Meat, g/100 BW	6.43	6.60	6.37	6.44	0.086	0.282
Cooking loss, %	20.6^b^	24.8^a^	25.2^a^	23.5^ab^	1.041	0.026
CIE L*	54.4	53.4	55.8	54.6	1.139	0.531
CIE a*	2.13	3.05	1.56	2.31	0.410	0.108
CIE b*	7.61	8.87	8.85	9.25	0.586	0.241
pH	6.23	6.17	6.23	6.14	0.062	0.653

^1^Values represent the mean of 7 replicates per treatment (*n* = 7/treatment)

^2^*CON* Non-challenged naïve control, *INFECT* Challenged control, *SAL* Challenged/salinomycin-added diet, *EO* Challenged/essential oil-added diet, *BW* Body weight, *SEM* Standard error of the means

^a-b^Means with different superscripted letters differ significantly (*P* < 0.05)

## Discussion

Avian coccidiosis is a devastating enteric disease caused by several species of *Eimeria* that results in huge economic losses. Several anticoccidial agents, including ionophores, have been added to poultry diets to control coccidiosis, but there are growing concerns over the emergence of drug-resistant oocysts and drug residues in the meats. Essential oil-based preparations have been marketed as an alternative natural anticoccidial, as they have multiple biological properties that directly inhibit the *Eimeria* spp. They also upregulate the host immune response, improve the antioxidant capacity, and exhibit antimicrobial activity upon ingestion [8]. We used the 20× of the manufacture’s recommended dose of the vaccine via gavage [17] and this reflects the farm conditions of *Eimeria* infection, which are typically infested with multiple *Eimeria* oocysts. The anticoccidial efficacy of *O. vulgare* and *Citrus* spp.-based essential oils [16] were determined in the vaccine-induced coccidiosis model.

In this study, inoculation with high doses of live coccidiosis vaccine at 14 d post-hatch did not impair growth performance of the chickens although BWG of the INFECT group was reduced by an average of 4.8% during the phase compared to that of the CON group. During the finisher phase, BWG in the INFECT vs. the CON group was 2.2% lower (on average), indicating recovery from the coccidiosis challenge with age. The difference in the FCR of the CON vs. the INFECT groups tended to be wider (1.2% to 3.5%) during the starter and finisher phases. Thus, the impact of the coccidiosis vaccine challenge on production performance persisted throughout the study period but was minimal. The lack of a challenge effect on performance may be related to the composition of the basal diet used in this study containing exogenous enzymes and probiotics or timing of challenge. This was intended to reflect the field situation, as commercial diets contain several feed additives, such as exogenous enzymes (e.g., phytase, NSPase, protease, or lipase), amino acids, vitamins, minerals, and medium-chain fatty acids. Dersjant-Li et al. [21] reported that a combination of exogenous enzymes and *Bacillus* spp.-based probiotics reduced the gut damage and performance loss caused by coccidial challenge. Dietary monolaurate, marketed as an alternative to antibiotics, played an important role in stimulating growth and improving meat quality when included in the diets of broiler chickens [22]. Finally, phytase supplementation to broiler chicken diets was equally effective at improving the growth performance with or without *Eimeria acervuline* challenge [23]. Thus, the negative performance effects of 25× the recommended dose of the coccidial vaccine [24] appear to be minimal in this study. The chickens fed with a diet containing anticoccidial SAL performed equal to the CON group, but EO-fed chickens performed lowest (i.e., FCR) in all treatment groups. These results indicate that dietary anticoccidial vs. natural alternatives is more effective at controlling avian coccidiosis. At this stage, it is not clear why dietary EO did not affect BWG of chickens following *Eimeria* vaccine challenge. Further studies are required to investigate whether the dietary EO mitigate avian coccidiosis induced by field-type *Eimeria* spp. or higher doses of the coccidiosis vaccine.

Chick’s exposure to *Eimeria* spp. impairs the host antioxidant defense mechanisms via *Eimeria*-induced gut damage that triggers intestinal oxidative stress – this is the area of action for in-feed antioxidants as natural anticoccidial agents [6]. Indeed, Gadde et al. [8] observed beneficial effects of EO preparations due to their well-known antioxidant properties. In this study, the *Eimeria* vaccine challenge induced oxidative stress as evidenced by decreased SOD activity in the serum, and the *Eimeria*-induced oxidative stress was partially mitigated by dietary SAL. Dietary EO did not improve the antioxidant capacity of the broiler chickens but tended to increase the SOD activity compared to that of the INFECT group. Idris et al. [25] found that EO with *in vitro* and *in vivo* antioxidant activities exhibited variable results for avian coccidiosis. Alpha-1-acid glycoprotein is a moderate acute-phase protein that is secreted from the hepatocytes in response to inflammation or infection. Increased levels of the acute-phase proteins, including alpha-1-acid glycoprotein, in the blood serve to restore the disturbed homeostatic conditions during healing [26]. However, this process negatively affects production performance, as elevated acute-phase protein levels require energy expenditure [27]. In line with the SOD activity patterns, the serum concentrations of alpha-1-acid glycoprotein were elevated by the *Eimeria* vaccine challenge but were mitigated by dietary SAL. The latter finding coincides with the increased antioxidant capacity of SAL and may explain the increased BWG in this group compared to the INFECT group. Interestingly, the dietary EO preparations further aggravated the *Eimeria*-induced increase in alpha-1-acid glycoproteins. How the dietary EO elevated the vaccine-challenged increase in acute-phase proteins is unclear and requires further investigation.

Giannenas et al. [28] reported that the coliform in the cecal contents of broiler chickens was altered by coccidiosis challenge. Macdonald et al. [29] noted that the amount of Enterobacteriaceae in the cecum increased, while the abundances of Bacillales and Lactobacillales decreased in chicks inoculated with *Eimeria tenella* (compared to a non-challenged control). Indeed, *Eimeria* infection marginally reduced the total VFA by 19% compared to that of the CON group; this is consistent with the observations of Leung et al. [30]. However, the addition of SAL or the EO preparation into the diets following *Eimeria* vaccine challenge did not affect the VFA concentrations in the cecum (though dietary EO preparation increased the BCFA contents). It is known that increased concentrations of VFA in the gut digesta can be considered beneficial, as they inhibit pathogenic bacteria invasion and aid enterocyte homeostasis [31]. On the other hand, excessive accumulation of BCFA contents noted in this study might indicate the abnormal scenario of fermentation by gut microbiota on the non-absorbed amino acids or proteins reaching the cecum. It is of interest to see that the increased concentration of BCFA contents in the EO group (see Table 5) is associated with highest FCR during d 7 to 20 (see Table 2). Thus, it might be likely that the increase in BCFA contents in the EO group is in part related to EO-mediated change in gut microbiota or protein digestion, especially at early days. However, as the portion of BCFA contents in total VFA is kept minimal, their contribution on gut health needs to be clarified.

In this study, the breast and thigh muscle meat qualities were analyzed on the 21st day post-*Eimeria* vaccine challenge (i.e., 35 d of age) to investigate if early exposure to *Eimeria* affects meat qualities. *Eimeria* challenge significantly increased the cooking loss of thigh meat compared to that of the CON group. In contrast to our results, Chodova et al. [32] reported that subclinical *E. tenella* infection did not affect the meat color and cooking loss of broiler chicken breast meat. Interestingly, the dietary EO preparation tended to lower the cooking loss of thigh meat compared to that of the INFECT group. Tauer et al. [33] reported that dietary oregano essential oil did not affect the pH of the breast meat but increased the meat colors when the broiler chickens were exposed to a coccidiosis disease challenge. The results of our study, supported by earlier work [32, 33], suggest that the meat quality of broiler chickens challenged by *Eimeria* warrants further investigation.

## Conclusions

In conclusion, dietary SAL vs. EO performed better in broiler chickens following coccidiosis vaccine challenge. Dietary EO preparation lowered the amounts of oocysts present in the litter and increased the VFA concentration in the cecal digesta. Further studies are required to evaluate the effect of EO preparations on gut health including gut barrier parameter and gut microbiota profile in experimental coccidiosis induced by pathogenic field-type *Eimeria* spp.

## Data Availability

The raw data for the current study are available from the corresponding author on reasonable request.

## Abbreviations

BCFA: Branched-chain fatty acid
BWG: Body weight gain
ELISA: Enzyme-linked immunosorbent assay
EO: Essential oil
FCR: Feed conversion ratio
SAL: Salinomycin
sIgA: Secretory immunoglobulin A
SOD: Superoxide dismutase
VFA: Volatile fatty acids
